# D-dopachrome tautomerase drives astroglial inflammation via NF-κB signaling following spinal cord injury

**DOI:** 10.1186/s13578-022-00867-7

**Published:** 2022-08-14

**Authors:** Hui Li, Bingqiang He, Xingyuan Zhang, Huifei Hao, Ting Yang, Chunshuai Sun, Honghua Song, Yingjie Wang, Yue Zhou, Zhenjie Zhu, Yuming Hu, Yongjun Wang

**Affiliations:** 1 Key Laboratory of Neuroregeneration of Jiangsu and Ministry of Education, Co-innovation Center of Neuroregeneration, Nantong University, 226001 Nantong, People’s Republic of China; 2 Department of Rehabilitation Medicine, Affiliated Hospital of Nantong University, 226001 Nantong, People’s Republic of China; 3 Key Laboratory of Neuroregeneration, Nantong University, 19 Qixiu Road, 226001 Nantong, People’s Republic of China

**Keywords:** D-DT, Spinal cord injury, Astrocyte, Inflammation, NF-κB, Central nervous system, MAPKs

## Abstract

**Background:**

Reactive astrocytes are increasingly recognized as crucial regulators of innate immunity in degenerative or damaged central nervous system (CNS). Many proinflammatory mediators have been shown to drive inflammatory cascades of astrocytes through activation of NF-κB, thereby affecting the functional outcome of the insulted CNS. D-dopachrome tautomerase (D-DT), a newly described cytokine and a close homolog of proinflammatory macrophage migration inhibitory factor (MIF), has been revealed to share receptor and overlapping functional spectrum with MIF, but little is known about its roles in the neuropathological progression of the CNS and relevant regulatory mechanisms.

**Results:**

D-DT protein levels were significantly elevated within neurons and astrocytes following SCI. Analysis of transcriptome profile revealed that D-DT was able to activate multiple signal pathways of astrocytes, which converged to NF-κB, a hub regulator governing proinflammatory response. Rat D-DT recombinant protein was efficient in inducing the production of inflammatory cytokines from astrocytes through interaction with CD74 receptor. Activation of mitogen-activated protein kinases (MAPKs) and NF-κB was observed to be essential for the transduction of D-DT signaling. Administration of D-DT specific inhibitor at lesion sites of the cord resulted in significant attenuation of NF-κB activation and reduction of the inflammatory cytokines following SCI, and accordingly improved the recovery of locomotor functions.

**Conclusion:**

Collectively, D-DT is a novel proinflammatory mediator of astrocytes following SCI. Insights of its cell-specific expression and relevant proinflammatory mechanisms will provide clues for the control of CNS inflammation.

**Supplementary Information:**

The online version contains supplementary material available at 10.1186/s13578-022-00867-7.

## Background

Astrocytes are the most abundant glial cell type in the central nervous system (CNS) that contribute to the tissue homeostasis by acting trophic, structural and metabolic roles [1, 2]. Injury or pathogenesis of the CNS, however, will results in morphological, molecular and functional changes of astrocytes that disrupt their homeostatic ability and make the profound effects on survival and function of other neural cells [2–5]. The activation of astrocytes in the pathological contexts is termed as astrocyte reactivity, whose biological importance is still in controversy [5–7]. For example, a reduction of reactive astrocytes during the early phases of CNS injury can lead to exacerbation of motor deficits, characterized by demyelination and neuronal death [8, 9], whereas in the chronic phase of experimental autoimmune encephalomyelitis (EAE), depletion of astrocytes is able to ameliorate clinical signs of the disease [10]. Regardless of those debates in the CNS disorders, the reactive astrocytes are consistently recognized as crucial regulators of innate immunity in the injured CNS [3, 5]. They augment the expression of pattern-recognition receptors (PRRs) following CNS injury, which are collectively known for their role in the activation of the innate immune system [11, 12]. In response to diverse inflammatory signals derived from many cell types including neurons and other glia, reactive astrocytes are able to secrete proinflammatory cytokines and chemokines that are detrimental to the functional outcomes of CNS, even though paired with the releases of several factors essential for neuronal survival [8, 13–18].

Damage-associated molecular patterns (DAMPs) that are passively released from necrotic nerve cells or actively secreted by immune cells are robust triggers of astrocytic inflammation by interaction with the PRRs on the plasma membrane [19–21]. Previous studies have shown that several DAMPs including HMGB1 and macrophage migration inhibitory factor (MIF) are efficient in promoting astrocytic production of proinflammatory cytokines and chemokines, and as a result, significantly impacting on the functional recovery of the pathological CNS [17, 18, 20, 22, 23]. Although being activated by various inflammatory mediators released in the lesion milieu, astrocytes can initiate innate immune responses through complex but also interwoven intracellular signal pathways [21, 24]. Of note, the mitogen-activated protein kinases (MAPKs)/NF-κB signaling is one of the most enriched pathways in regulation of astrocytic inflammation [16, 18, 22, 24]. In fact, selective inhibition of NF-κB signaling in astrocytes has been verified to play protective roles after spinal cord injury (SCI) by increasing axonal sparing and sprouting [14, 15]. Interestingly, HMGB1 is found to be inefficient in activating MAPKs/NF-κB pathway of astrocytes to mediate the production of prominent proinflammatory cytokines such as TNF-α and IL-1β, due to absence of MyD88 adaptor downstream of Toll-like receptors (TLRs) [1, 20, 25–27]. Therefore, reactive astrocytes can be defined as a specific inflammation-associated cell population with differential genomic programs to those of microglia.

D-dopachrome tautomerase (D-DT) is a homolog of MIF with low but significant sequence and structural similarity [28, 29]. Comparatively, MIF is well studied and recognized as a potent proinflammatory mediator besides its other physiological and pathological functions [30–32]. MIF has been constitutively or inducibly observed within neurons, microglia, astrocytes, ependymal cells and epithelial cells of the choroid plexus in the CNS, and sustaining a high protein level through autocrine/paracrine release at the lesion sites [32, 33]. As one of crucial DAMPs, it mediates neurological disorders by activating microglia and astrocyte inflammation through binding with CD74, the component of MIF receptor complex [18, 34, 35], or with CXCR2/4 receptor [36]. Although D-DT shares the receptor and biological functions with its homolog MIF in a certain degree [16, 18, 29, 37], the cytokine also displays distinct pathophysiological roles due to lack of a pseudo (E)LR domain in association with mediating MIF’s binding with CXCR2 receptor, and the CXXC redox motif present on MIF [28, 37]. Direct evidence comes from that MIF positively, while DDT negatively contributes to adipose tissue inflammation [38]. To date, little is known about the specific roles of D-DT in regulation of tissue inflammation and the underlying mechanisms, especially in the pathological CNS. Deciphering the exact effects of D-DT on activating the astrocytic inflammation will provide a novel target for therapy of inflammation-related CNS disorder. In the present study, the expression of D-DT protein was examined in the injured spinal cord of rat, and its effects on the inflammatory activation of astrocytes, as well as the underlying mechanism were further investigated. Our results prove that inhibition of D-DT-mediated astrocytic inflammation following SCI will be beneficial for the functional recovery of the locomotor.

## Results

### SCI significantly induces expression of D-DT within specific cell types at lesion sites

**Fig. 1:**
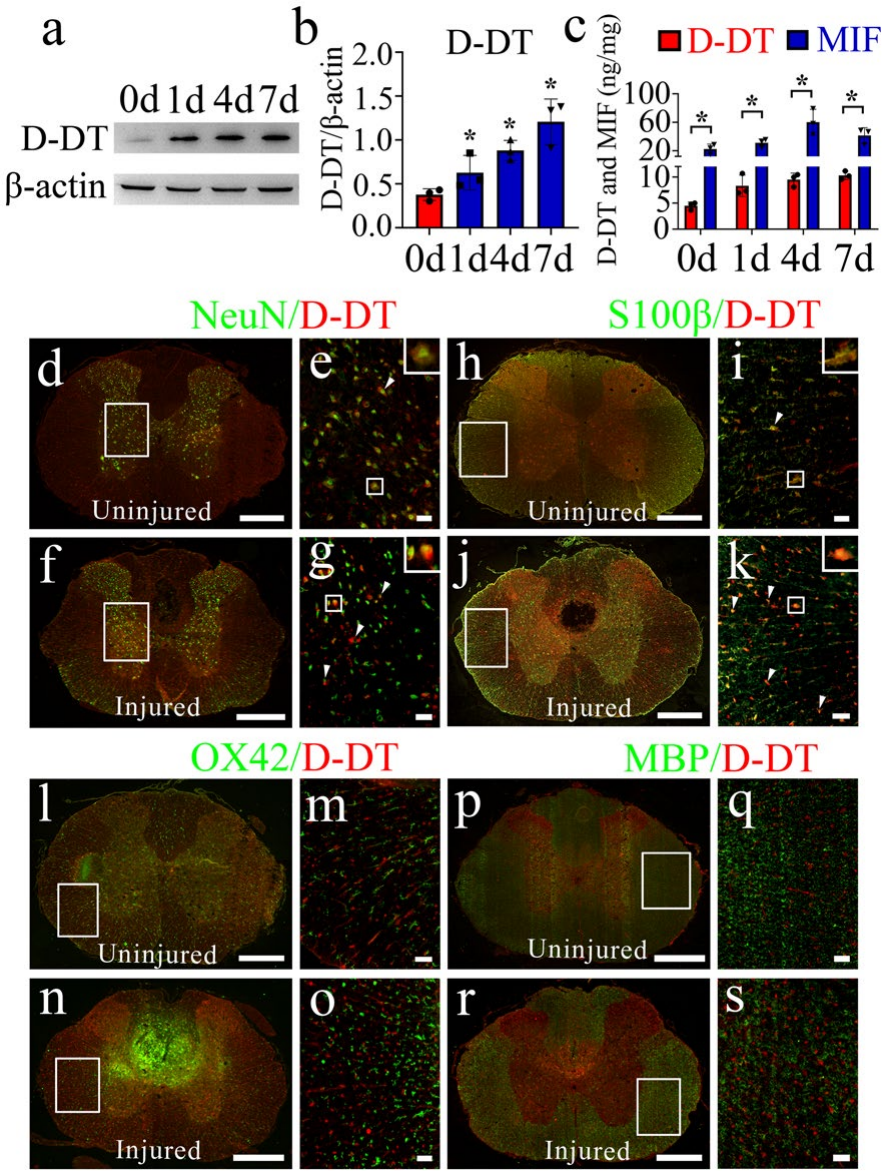
Examination of D-DT expression changes at lesion sites following rat SCI. **a** Western blot analysis of D-DT following cord contusion at 0d, 1d, 4d and 7d, respectively. **b** Quantification data as shown in **a**. Quantities were normalized to endogenous β-actin. **c** ELISA measurement of D-DT and MIF protein levels at lesion sites following SCI at 0d, 1d, 4d and 7d, respectively. **d-s** Immunostaining showed colocalization of D-DT with NeuN-positive neurons (**d-g**) and S100β-positive astrocytes (**h-k**), rather than with OX42-positive microglia (**l-o**) or MBP-positive oligodendrocytes (**p-s**) before or after SCI at 4d. Rectangle indicates region magnified. Arrowheads indicate the positive signals. Scale bars, 500 μm in (**d**), (**f**), (**h**), (**j**), (**l**), (**n**), (**p**) and (**r**); 50 μm in (**e**), (**g**), (**i**), (**k**), (**m**), (**o**), (**q**) and (**s**). Experiments were performed in triplicates. Error bars represent the standard deviation (*P < 0.05)

To understand SCI-induced dynamic changes of D-DT expression which is potentially associated with neuropathological progression, the protein levels of D-DT at lesion sites were determined at 0d, 1d, 4d and 7d following rat spinal cord contusion. Western blot demonstrated that SCI significantly induced the production of D-DT at various time points (Fig. 1a, b), with a similar inducible profile to those of other DAMPs [18, 20]. Parallel determination of MIF and D-DT protein levels by ELISA displayed that injury-induced D-DT protein at lesion sites was far less than MIF (Fig. 1c), indicating the differential effects of the two homologs on the neuropathology after SCI.

We next sought to examine the specific cell types of D-DT expression induced by SCI. The tissue sections were made from a 0.25 cm-length to the epicenter of contusion. Immunostaining revealed that D-DT protein colocalized with NeuN-positive neurons (Fig. 1d–g), and S100β-positive astrocytes (Fig. 1h-k), rather than with OX42-positive microglia (Fig. 1l-o) or MBP-positive oligodendrocytes (Fig. 1p-s) before or after SCI. The data indicate that the neurons and astrocytes are crucial cell sources of D-DT production following SCI.

### Transcriptome profile reveals that D-DT drives inflammatory signal pathways of astrocytes as a proinflammatory mediator

**Fig. 2:**
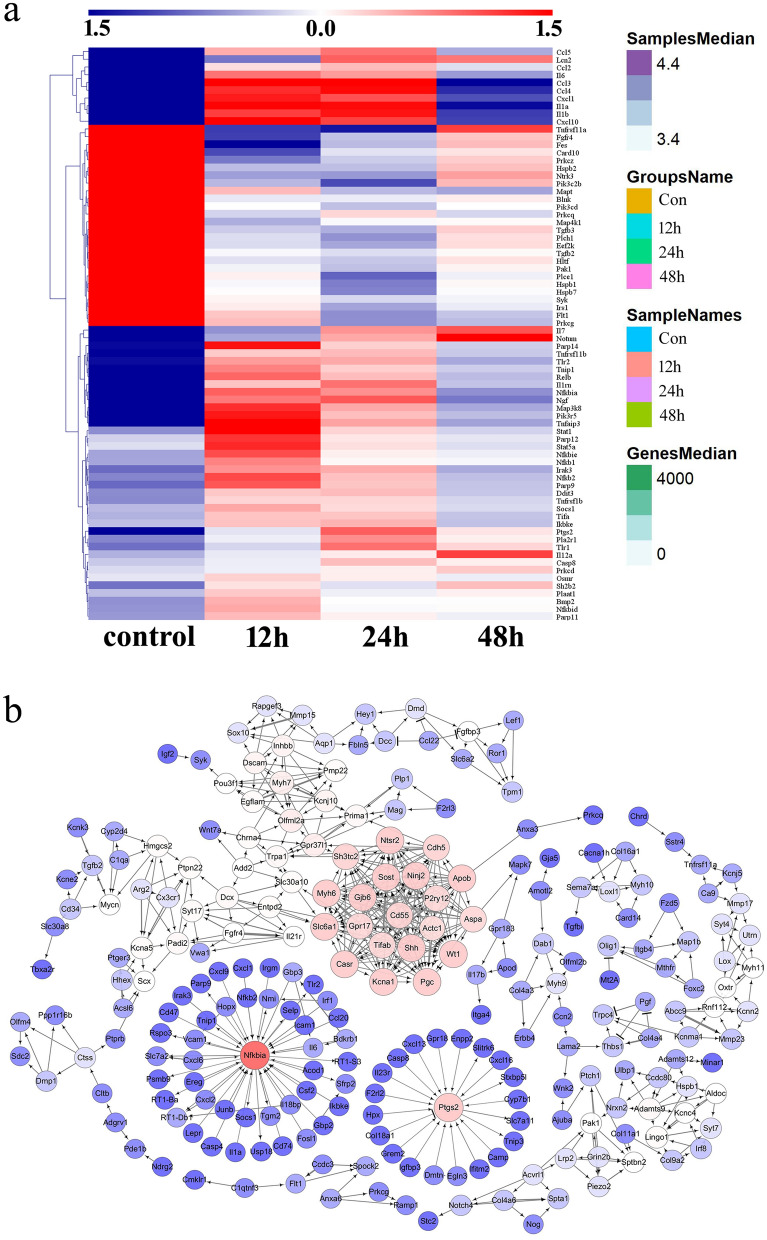
Expression profiling and gene network analysis of integrated DEGs in the astrocytes following stimulation with 1 µg/ml recombinant D-DT protein for 12, 24, and 48 h, respectively. **a** Heatmap and cluster dendrogram of integrated DEGs at 12, 24, and 48 h. The color scale shown at the top illustrates the relative expression level of the indicated mRNA across all samples: red denotes expression > 0 and blue denotes expression < 0. **b** A reconstructed gene network was created using the IPA on the basis of integrated DEGs

Despite several findings have suggested an involvement of D-DT in modulating cancer pathogenesis [39], improving glucose intolerance in obesity [40], protecting against heart failure [41], and exacerbating inflammation in sepsis [37], the physiological actions of DDT in the CNS have not been fully elucidated so far. To examine the exact effects made by D-DT on the astrocytes, the primary astrocytes isolated from spinal cord with purity over 95% were stimulated with 1 µg/ml recombinant D-DT protein for 12, 24, and 48 h, respectively (Additional file 1: Fig S1a). Analysis of transcriptome profile revealed that a total of 3391, 4746, and 2885 differentially expressed genes (DEGs) were identified at the 3 time points, with defined criteria of P < 0.05 and a greater or less than twofold changes (Additional file 1: Fig S1b). Integration of these DEGs characterized 1827 functional genes that were dynamically regulated by D-DT (Additional file 1: Fig S1c). KEGG enrichment revealed that these integrated DEGs were associated with TNF-α, cytokine-cytokine receptor interaction, NF-κB, and MAPKs signaling pathways (Additional file 1: Fig S1d), which were responsible for mediating several important biological processes including wound healing, response to IL-1, IFN-γ and LPS, ECM organization and gliogenesis, as analyzed by GO enrichment (Additional file 1: Fig S1e). The data unveiled the proinflammatory role of D-DT, along with other biological functions on the astrocytes at molecular levels, The dynamic changes of DEGs involved in regulating inflammatory responses were illustrated by heatmap and cluster dendrogram (Fig. 2a). To identify the key regulators implicated in D-DT-induced inflammatory activation of astrocytes, the ingenuity pathway analysis (IPA) was further performed for the DEGs integrated at 12, 24 and 48 h. A reconstructed gene network revealed that NF-κB was highlighted as the crucial regulator with the highest weight value in response to D-DT stimulation (Fig. 2b). The transcriptome profile infers that D-DT may act as proinflammatory mediator of astrocytes through activation of NF-κB.

### D-DT promotes the astrocytic production of inflammatory cytokines through CD74 receptor

**Fig. 3:**
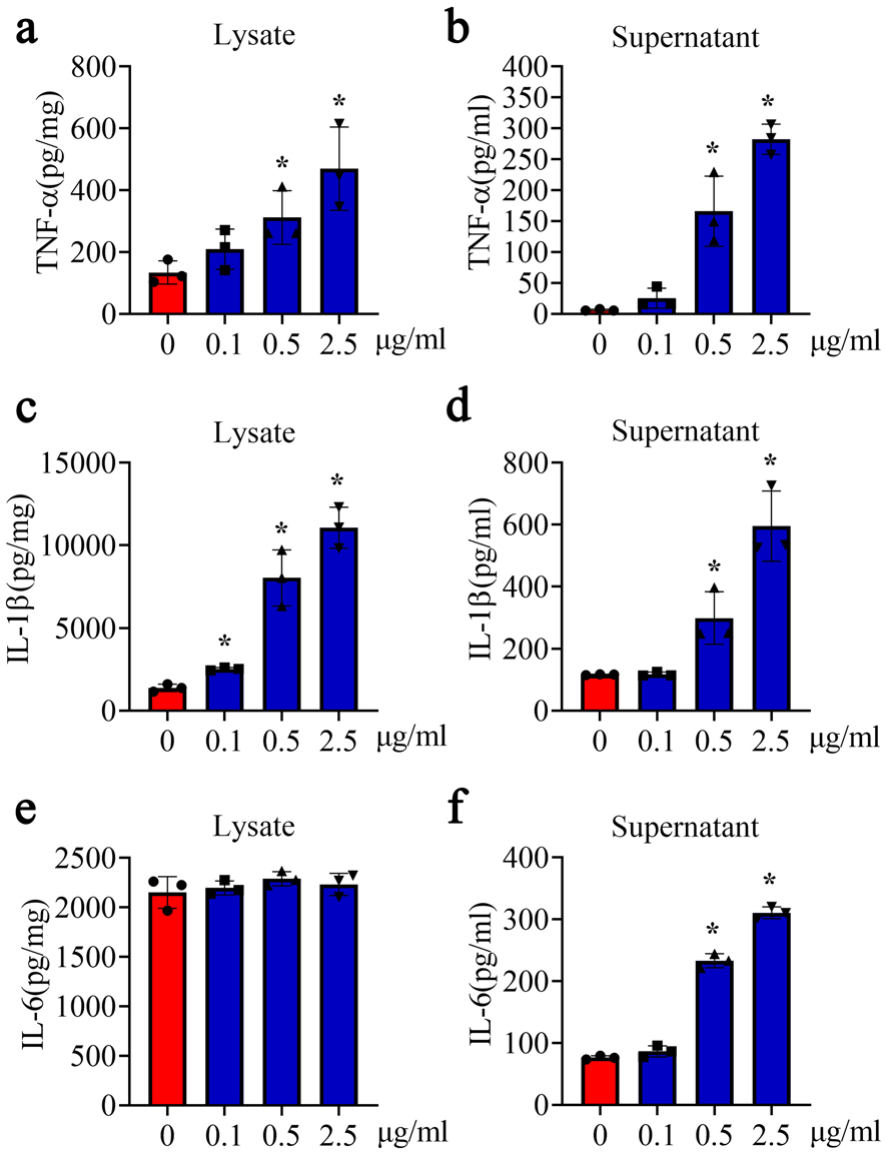
Determination of D-DT-induced inflammatory cytokines from astrocytes. The supernatants and lysates of astrocytes were determined by ELISA for the inflammatory cytokines TNF-α (**a**, **b**), IL-1β (**c**, **d**) and IL-6 (**e**, **f**) after cell stimulation with 0-2.5 µg/ml recombinant D-DT protein for 24 h. Experiments were performed in triplicates. Error bars represent the standard deviation (*P < 0.05)

**Fig. 4:**
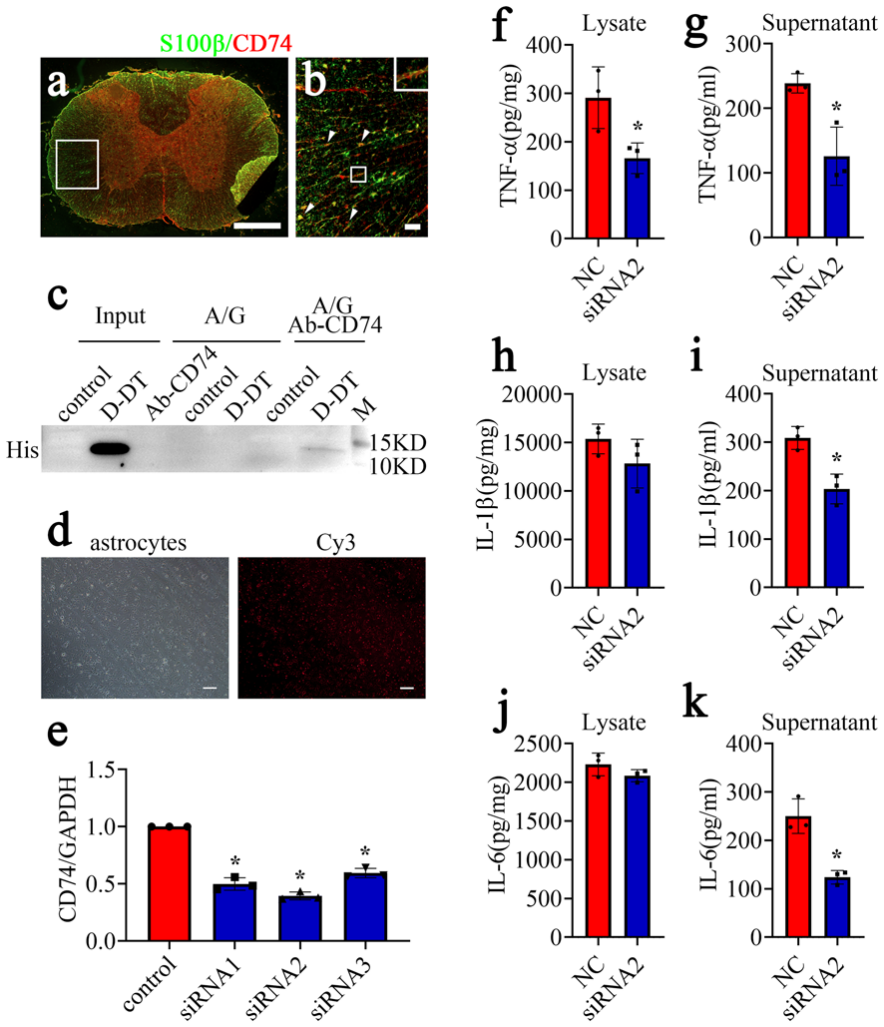
D-DT promoted astrocytic production of inflammatory cytokines through interaction with CD74 receptor. **a**, **b** Immunofluorescence showed colocalization of CD74 with S100β-positive astrocytes in the spinal cord. Rectangle indicates region magnified. Arrowheads indicate the positive signals. **c** Binding assay of D-DT with CD74 receptor in the primary astrocytes. The immunoprecipitation was performed with anti-CD74 antibody (Ab-CD74), followed by detection of the components of the CD74-associated complexes with anti-His antibody. The control meant that the astrocytes were stimulated with 0.01 M PBS, and the D-DT indicated that the astrocytes were stimulated with 1 µg/ml recombinant D-DT protein with N-terminal His-tag. **d** Determination of siRNA transfection efficiency by Cy3 control. **e** Interference efficiency of three siRNA oligonucleotides for CD74 was measured by RT-PCR, and siRNA2 was used for the knockdown experiments. **f-k** Determination of TNF-α (**f, g**), IL-1β (**h**, **i**) and IL-6 (**j**, **k**) by ELISA from astrocytes following CD74 knockdown for 48 h and subsequent stimulation with 1.0 µg/ml recombinant D-DT protein for 24 h. Scrambles were used as control. Scale bars, 500 μm in (**a**), 50 μm in (**b**) and (**d**). Experiments were performed in triplicates. Error bars represent the standard deviation (*P < 0.05)

To substantiate the proinflammatory roles of D-DT on astrocytes, the primary cells were stimulated with rat recombinant D-DT protein at concentration of 0–2.5 µg/ml for 24 h. ELISA measurement of the cell supernatants and lysates showed that the production of TNF-α, IL-1β and IL-6 was inducibly increased with D-DT-concentration dependence (Fig. 3a–f). As D-DT induces leukocyte signaling and effector via binding to CD74 receptor with high affinity [37], we also interrogated the signal axis in the astrocytes. Tissue immunostaining demonstrated that CD74 receptor was detected in the S100β-positive astrocytes of the spinal cord (Fig. 4a, b). Co-immunoprecipitation was subsequently carried out using anti-CD74 antibody. As shown in Fig. 4c, recombinant His-tagged D-DT was present in the CD74-associated complexes immunoprecipitated with anti-CD74 antibody. The results indicate that D-DT can bind with CD74 surface receptor of astrocytes.

To address whether the ability of D-DT to activate inflammation of astrocytes is strictly dependent on the presence of the receptor CD74, we knocked down CD74 receptor and observed the expression changes of inflammatory cytokines in the astrocytes. The siRNA oligonucleotide (siRNA2) with nearly 60% knockdown efficiency of CD74 was chosen for interference (Fig. 4d, e). The CD74 receptor of astrocytes was knocked down by siRNA2 for 48 h, followed by the cell treatment with 1.0 µg/ml recombinant D-DT protein for 24 h. ELISA results displayed that the protein levels of TNF-α, IL-1β and IL-6 were remarkably reduced in the supernatants and/or lysates following CD74 interference (Fig. 4f–k). The data indicate that D-DT potentiates astrocyte inflammation through CD74 receptor.

### Extracellular D-DT activates MAPKs/NF-κB pathway of astrocytes via CD74 receptor

**Fig. 5:**
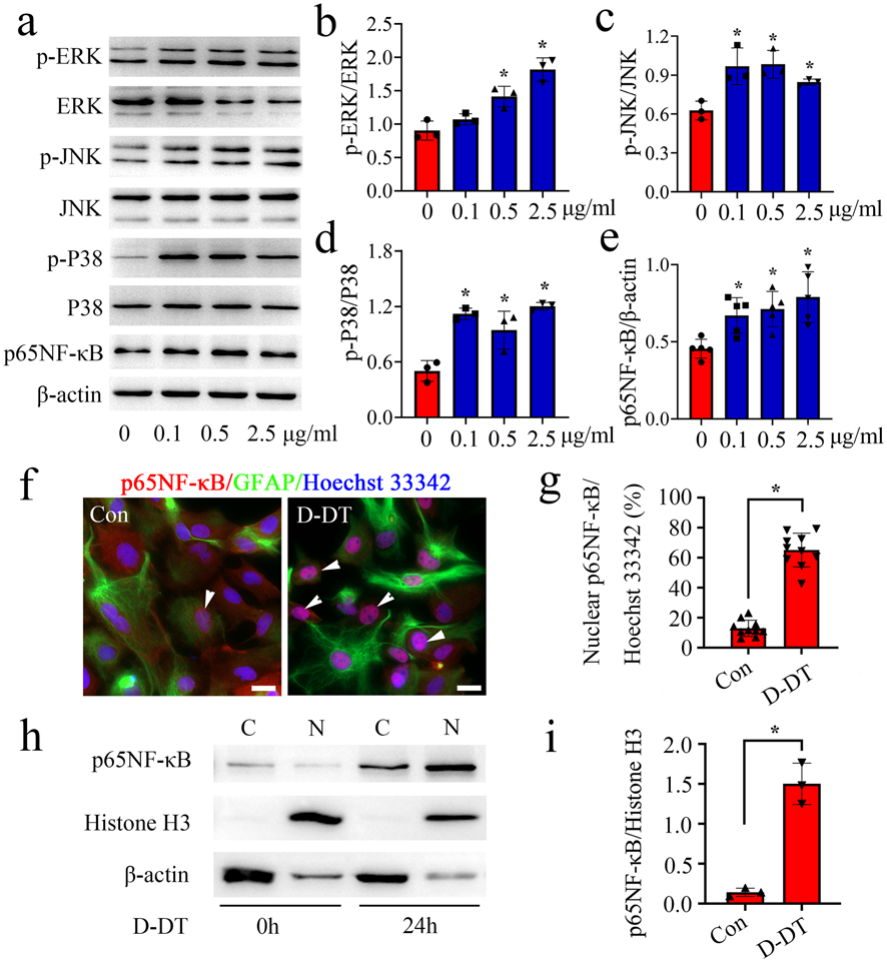
Effects of D-DT on the activation of intracellular MAPKs/NF-κB signaling in the astrocytes. **a** Western blot analysis of phosphorylation of ERK, P38, JNK kinase and p65NF-κB protein after stimulation of astrocytes with 0-2.5 µg/ml recombinant D-DT protein for 24 h. **b–e** Quantification data as shown in **a**.** f** Immunofluorescence showed the distribution of p65NF-κB in the cytoplasm and nucleus of astrocytes following treatment with 1 µg/ml recombinant D-DT for 24 h. Arrowheads indicate p65NF-κB-positive nucleus. **g** Quantification of p65NF-κB-positive nucleus co-stained with Hoechst 33,342 as shown in **f**. **h** Western blot analysis of p65NF-κB protein levels in the cytoplasm and nucleus following astrocytes treatment with 1 µg/ml recombinant D-DT for 24 h. **i** Quantification data as shown in **h**. Quantities were normalized to endogenous β-actin (cytoplasm) or Histone H3 (nucleus). Scale bar, 20 μm in **f**. Experiments were performed in triplicates. Error bars represent the standard deviation (*P < 0.05)

**Fig. 6:**
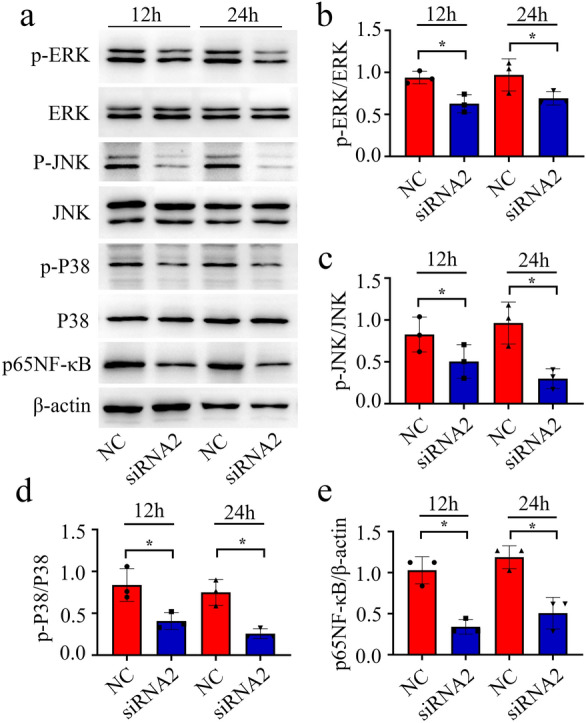
Effects of knockdown of CD74 receptor on the D-DT-mediated intracellular activation of MAPKs/NF-κB signaling in the astrocytes. **a** Western blot analysis of phosphorylation of ERK, P38, JNK kinase and p65NF-κB protein after siRNA2 knockdown of CD74 receptor for 48 h, prior to stimulation with 1 µg/ml recombinant D-DT protein for 12 and 24 h, respectively. **b–e** Quantification data as shown in **a**. Quantities were normalized to endogenous β-actin. Experiments were performed in triplicates. Error bars represent the standard deviation (*P < 0.05)

As MAPKs/NF-κB signaling is a prominent inflammatory pathway of astrocytes [14, 15], we thus proceed to observe the phosphorylated activation of components of MAPKs/NF-κB pathway in the astrocytes following stimulation with rat recombinant D-DT. Results revealed that exposure of astrocytes to 0-2.5 µg/ml D-DT for 24 h was efficient in promoting phosphorylation of ERK, P38, JNK protein kinase and the expression of p65NF-κB protein (Fig. 5a–e). Immunostaining of p65NF-κB demonstrated that D-DT was able to facilitate the nuclear translocation of this transcription activator (Fig. 5f, g). Immunoblot analysis of cytosolic and nuclear fraction also substantiated that D-DT markedly increased the protein abundance of p65NF-κB in the nucleus of astrocytes other than in the cytoplasm (Fig. 5h, i). Knockdown of CD74 expression with siRNA2 significantly attenuated the phosphorylated effects of D-DT on these proteins at 12 and 24 h (Fig. 6). The data indicate that the proinflammatory D-DT protein is able to activate MAPKs/NF-κB pathway of astrocytes via CD74 receptor.

### D-DT induces inflammatory response of astrocytes through activation of MAPKs/NF-κB pathway

**Fig. 7:**
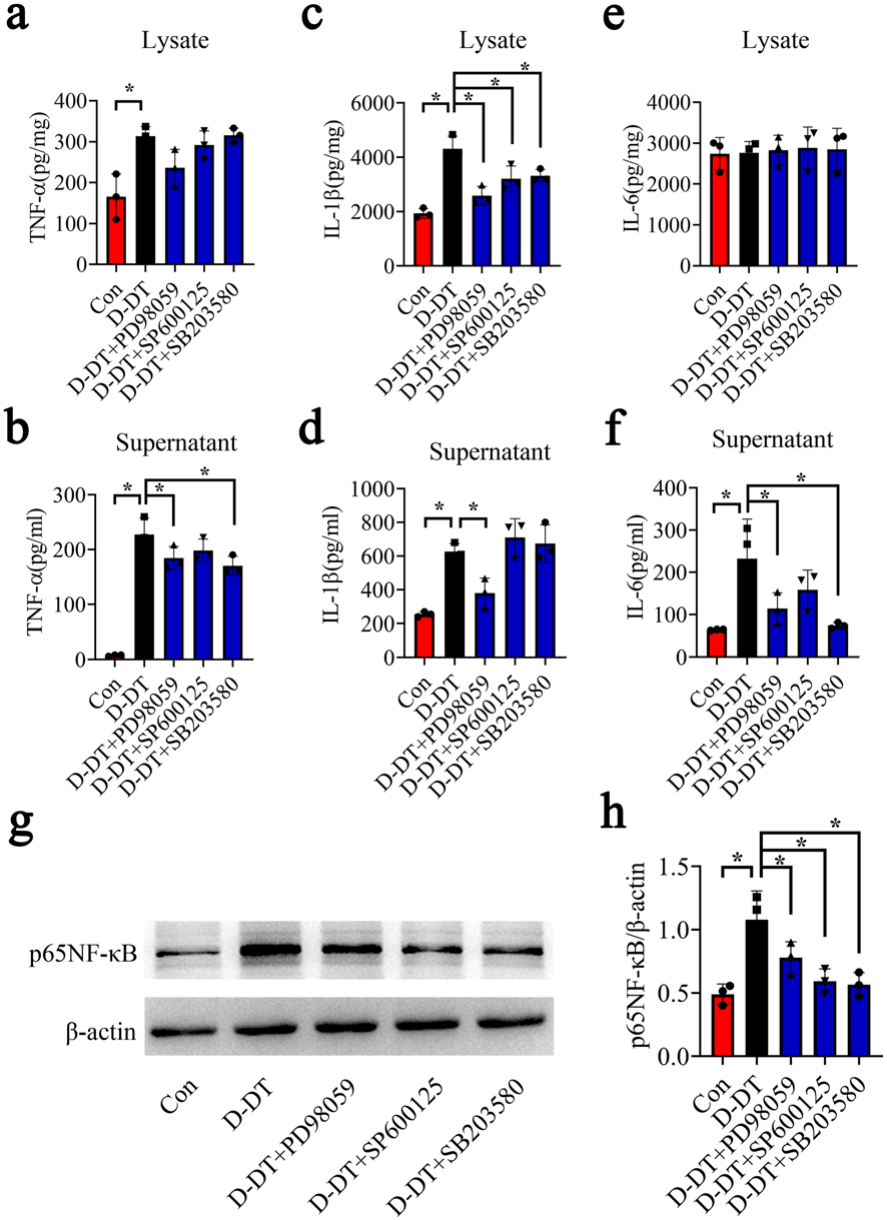
Inhibition of MAPKs attenuated D-DT-induced production of inflammatory cytokines from astrocytes. **a–f** ELISA determination of TNF-α (**a, b**), IL-1β (**c, d**) and IL-6 (**e, f**) in supernatant and lysate following astrocytes treatment with 1 µg/ml recombinant D-DT in the presence of 10 µM P38 (SB203580), 10 µM JNK (SP600125), or 10 µM ERK (PD98059) inhibitor for 24 h. **g, h** Western blot analysis of p65NF-κB protein levels following astrocytes treatment with 1 µg/ml recombinant D-DT in the presence of 10 µM P38 (SB203580), 10 µM JNK (SP600125), or 10 µM ERK (PD98059) inhibitor for 24 h. Experiments were performed in triplicates. Error bars represent the standard deviation (*P < 0.05)

To clarify whether D-DT-mediated activation of MAPKs/NF-κB pathway is involved in the production of inflammatory cytokines in astrocytes, the selective inhibitor of ERK (PD98059), JNK (SP600125) or P38 (SB203580) was applied to treat primary astrocytes in the presence of D-DT. Results demonstrated that addition of 10 µM inhibitor of PD98059, SP600125 or SB203580 to the culture in the presence of 1 µg/ml recombinant D-DT protein for 24 h, was able to decrease the production of TNF-α, IL-1β and IL-6 in the astrocytes (Fig. 7a–f). Accordingly, the activation of p65NF-κB was also remarkably attenuated by the inhibitors (Fig. 7g, h). The data indicate that D-DT-mediated activation of MAPKs/NF-κB pathway contributes to astrocytic inflammation.

### Administration of D-DT selective inhibitor significantly inactivates NF-κB and the downstream inflammatory cytokines following SCI

**Fig. 8:**
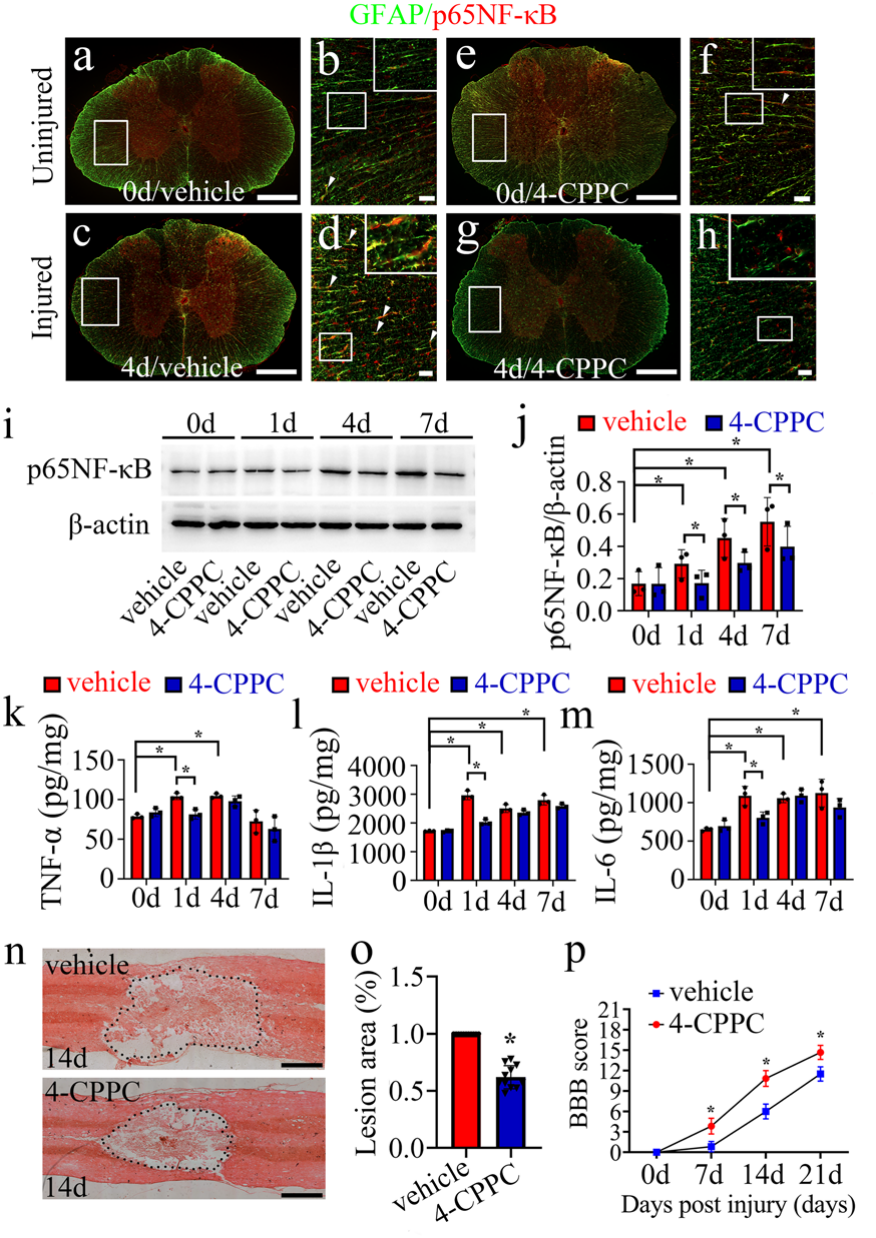
D-DT inhibitor attenuates NF-κB signaling and ameliorates locomotor function following rat SCI. **a–h** Immunostaining showed colocalization of p65NF-κB with GFAP-positive astrocytes following injection of 8 µl of 100 mM vehicle or 4-CPPC at lesion sites of the contused cord at 0d and 4d. Rectangle indicates region magnified. Arrowheads indicate the positive signals. (**i**) Western blot analysis of p65NF-κB at lesion sites following treatment with vehicle or 4-CPPC inhibitor at 0d, 1d, 4d and 7d. **j** Quantification data as shown in (**i**). Quantities were normalized to endogenous β-actin. **k–m** ELISA assay of TNF-α (**k**), IL-1β (**l**) and IL-6 (**m**) following cord treatment with vehicle or 4-CPPC inhibitor at 0d, 1d, 4d, and 7d, respectively. **n** HE staining of the injured spinal cord at 14 d after injection of 8 µl of 100 mM vehicle or 4-CPPC inhibitor at lesion sites. **o** Quantification data as shown in **n**. **p** BBB score of hindlimb at 0d, 7d, 14d and 21d following intrathecal injection of 8 µl of 100 mM 4-CPPC or vehicle at the lesion sites, n = 6. Scale bars, 500 μm in (**a**), (**c**), (**e**), (**g**) and (**n**); 50 μm in (**b**), (**d**), (**f**) and (**h**). Experiments were performed in triplicates. Error bars represent the standard deviation (*P < 0.05)

To elucidate whether inhibition of D-DT can attenuate NF-κB signaling following SCI, 8 µl of 100 mM vehicle or D-DT inhibitor 4-CPPC was intrathecally injected at the lesion sites. Immunostaining demonstrated that SCI-induced increase of p65NF-κB in the astrocytes was significantly inhibited by 4-CPPC (Fig. 8a–h). Western blot also showed that the protein levels of p65NF-κB at lesion sites were markedly decreased by 4-CPPC treatment at 1d, 4d and 7d, comparing with the vehicle (Fig. 8i, j). Accordingly, measurement of inflammatory cytokines by ELISA displayed that SCI-induced production of TNF-α, IL-1β and IL-6 from 1d onwards was significantly reduced by 4-CPPC administration at 1d (Fig. 8k–m). Taken together, inhibition of D-DT following SCI is able to suppress astrocyte-mediated activation of NF-κB and the production of downstream inflammatory cytokines.

### Inhibition of D-DT ameliorates motor function following rat SCI

To understand the effects of deficient D-DT on the functional recovery following SCI, 8 µl of 100 mM vehicle or 4-CPPC was injected at lesion sites as above. Morphological observation of the HE sections revealed that 4-CPPC treatment significantly reduced the lesion size of the cord in comparison with the vehicle at 14d following contusion (Fig. 8n, o). BBB scores were further assessed during 3 weeks to evaluate functional effects of D-DT inhibition. Behavioral tests showed that 4-CPPC treatment significantly improved the recovery of hindlimb locomotor function of the spinal cord contused rats (Fig. 8p). The data indicate that inhibition of D-DT is able to ameliorate locomotor function following rat SCI.

## Discussion

D-DT was first described as an enzyme which catalyzes the tautomerization and decarboxylation of the substrate D-dopachrome to 5, 6-dihydroxyindole [42]. For many years, the physiological role of D-DT remains an intriguing enigma, due to that D-dopachrome does not exist in mammals [29]. Growing evidence has shown that D-DT is relatively highly expressed in multiple tissues including liver, heart, brain, spleen, lung, skeletal muscle, kidney, and testes [42, 43]. The well characterized biological functions of D-DT are conferred to its proinflammatory activity in sepsis [37] and favoring for tumorigenesis [39, 44], the properties shared with MIF. Of note, D-DT also plays apposed physiological functions to those of MIF, ranging from chemotactic activities in recruiting monocytes and leukocytes [28, 36], adipogenesis [45, 46], wound healing [47], and ischemic injury of heart [41]. To date, less information is available about the pathophysiological significance of D-DT in regulation of CNS disorder, especially for its impacts on the injured cord [16]. In the present study, we unveiled that MIF homolog D-DT participated in the neuropathological progression by serving as proinflammatory mediator of astrocytes following SCI. The results provide a novel molecular target for interference of the CNS inflammation.

The expression pattern of two MIF homologs in the spinal cord has been observed to be slightly different, as MIF is constitutively or inducibly expressed within neurons, astrocytes, microglia and other CNS-resident cell types [48], whereas D-DT is only detectable within neurons and astrocytes. These imply that MIF might be more sensitive or efficient than D-DT in serving as alarmins or DAMPs of CNS. Unexpectedly, D-DT is absent from microglia before or after SCI, even though it retains conserved gene structure and genomic linkage as those of MIF [28]. The deficient expression of D-DT in microglia may attribute to the higher threshold of DAMPs in inducing D-DT than MIF production, because an equivalent stimulation of LPS in macrophages has been shown to result in 20-fold higher levels of MIF than its homolog [37]. Of course, it cannot be excluded that other regulatory mechanisms may contribute to the differential expression patterns of the two homologs. We herein suggest that the resident microglia of the spinal cord will not be the cell source of D-DT when considering for intervention of the CNS inflammation with genetic approaches.

NF-κB is one of key transcription factors that regulate many genes with determinant roles in the processes of immunity and inflammation [49, 50]. Reactive astrocytes that play critical roles in the neuropathological progression following SCI are regulated by NF-κB, as they synthesize and release pro-inflammatory mediators through the sustained activation of the protein [14, 15]. Selective inhibition of NF-κB in astrocytes is able to reduce the expression of proinflammatory chemokines and cytokines, as well as chondroitin sulfate proteoglycans associating with the formation of the glial scar, but leads to increased axonal sparing and sprouting beneficial for the functional recovery [14, 15]. Therefore, NF-κB, together with its activators, is the crucial player of astrocyte-mediated inflammation following SCI. In the present study, the result showed that the application D-DT inhibitor 4-CPPC in vivo at lesion site following SCI decreased the expression of p65NF-κB and inflammatory cytokines but did not show strong effects, especially in inflammatory cytokines. The main reason is that SCI triggers a variety of DAMPs, such as HMGB1, MIF and other proinflammatory mediators, to be actively secreted from leukocytes or passively released from necrotic cells. All of them are able to facilitate inflammation through activation of NF-κB [32, 51, 52]. The specific inhibitor of D-DT can only perform on inhibiting D-DT-mediated inflammatory responses in the injured spinal cord. Its inhibitory effects on inflammation were partially masked by other proinflammatory mediators. In short, D-DT has been identified to activate inflammation of astrocytes through MAPKs/NF-κB pathway, manifesting a novel proinflammatory mediator with the conserved regulatory mechanism in the CNS.

Traumatic injury of spinal cord is always followed by secondary tissue damages, characterized by the expansion of tissue damage from the lesion epicenter [53]. Many factors including excessive inflammation contribute to such neuropathological process which lasts for several weeks [54]. The magnitude of inflammation induced by endogenous and exogenous DAMPs has been found to well correlate with the extent of secondary injury [55]. Several evidences favor that early neuroinflammation is detrimental for functional recovery of the injured spinal cord [56, 57]. And what’s more, the early neuroinflammation in SCI is regarded to be CNS-derived [55]. D-DT-mediated astrocyte inflammation following SCI occurred within 24 h, and inhibition of D-DT activity has been shown to significantly promote the recovery of rat hindlimb locomotor function. Our results not only indicate an involvement of astrocyes in the early neuroinflammation, but also recapitulate the importance for intervention of early neuroinflammation following SCI.

## Conclusion

The expression of D-DT was significantly induced within the neurons and astrocytes following SCI. The cytokine was identified to be an active player in facilitating astrocytic inflammation through regulation of MAPKs/NF-κB pathway, by which exacerbated the lesion milieu of the cord. Inhibition of D-DT activity was efficient in attenuating NF-κB signaling and reducing the production of the inflammatory cytokines, thereby contributing to the functional recovery of the injured spinal cord.

## Methods

### Animals

Adult male Sprague-Dawley (SD) rats, weighing 180–220 g, were provided by the Center of Experimental Animals, Nantong University. All animal experiments were approved by *the Animal Care and Use Committee of Nantong University* and the *Animal Care Ethics Committee of Jiangsu Province*. All rats were housed in standard cages (five rats in each cage) in an environment maintained at 22 ± 2℃ on a 12–12 h light-dark cycle and had free access to water and food.

### Establishment of contusion SCI rat model and drug treatment

The number of animals subjected to surgery was calculated by six per experimental group in triplicate. The contusion SCI rat model was prepared as the previous description [58]. In a nutshell, all animals were anesthetized with 10% chloral hydrate (300 mg/kg) administered intraperitoneally. The fur around the surgical site was shaved and the skin was disinfected with iodophor. Then the spinous processes of T8-T10 vertebrae were surgically exposed, and a laminectomy was performed at the ninth thoracic vertebral level (T9) with the dura remaining intact. The exposed spinal cord segment (about 3 mm in length) received a 150-kilodyne contusion injury using the IH-0400 Impactor (Precision Systems and Instrumentation) injury device. The impact rod was removed immediately, and the wound was irrigated. For drug delivery, 8 µl of 100 mM D-DT inhibitor 4-CPPC (AOBIOUS) was slowly injected intrathecally, prior to the incision suture. The rats were subcutaneously administered with 0.2 ml antibiotics following surgery. Manual expression of bladders was performed twice a day until animals recovered spontaneous voiding.

### Cell culture and treatment

Astrocytes were prepared from the spinal cord of newborn SD rats, 1–2 days after birth, and the astrocytes were isolated and cultured according to previously described methods [17]. Briefly, the spinal cords removed from the spinal canal were placed into 0.01 M PBS containing 1% penicillin-streptomycin. The spinal cord capsule was stripped clean under the microscope, followed by mincing with scissors and digestion with 0.25% trypsin for 15 min at 37 ℃. Digestion was terminated by addition of Dulbecco’s Modified Eagle’s Medium - high glucose medium containing 10% fetal bovine serum (FBS), 1% penicillin-streptomycin and 1% L-glutamine. The suspension was then centrifuged at 1200 rpm for 5 min and the cells were resuspended and seeded onto poly-L-lysine pre-coated culture flask in the presence of 5% CO_2_. The medium was changed every 3 days until the whole flask is covered with cells. After 7–9 days, the culture flask was shaken at 250 rpm overnight to remove non-astrocytes. Astrocyte phenotype was evaluated by cell exhibiting a characteristic morphology and positive staining for the astrocytic marker glial fibrillary acid protein (GFAP). Astrocytes with purity more than 95% are acceptable for subsequent experiments.

To determine the effects of the selective inhibitor SB203580 (P38), SP600125 (JNK) or PD98059 (ERK) on the D-DT-induced astrocyte production of inflammatory cytokines, the cells were treated with 1 µg/ml recombinant D-DT (Aviva Systems Biology) in the presence or absence of 10 µM SB203580 (TOCRIS), 10 µM SP600125 (TOCRIS) or 10 µM PD98059 (TOCRIS) for 24 h prior to assay.

For knockdown of CD74 expression in the astrocytes, the cells were cultured on the six-well plates for 24 h, followed by transfection of CD74 siRNA2 (sense strand 5’-CAG GAU AUG GGC CAA AUG U dTdT-3’, antisense strand 5’-A CAU UUG GCC CAU AUC CUG dTdT-3’) or scramble siRNA (sense strand 5’-GGC UCU AGA AAA GCC UAU GC dTdT-3’, antisense strand 5’-GC AUA GGC UUU UCU AGA GCC dTdT-3’) with iMAX transfection reagent (Invitrogen) for 24 h. The astrocytes were subsequently incubated at medium in the absence of 1% penicillin-streptomycin for 24 h, and then stimulated by 1 µg/ml D-DT recombinant protein for another 12 and 24 h before ELISA or Western blot assay.

### Western blot

Protein was harvested from cells with a buffer containing 50 mM Tris (pH 7.4), 150 mM NaCl, 1% Triton X-100, 1% sodium deoxycholate, 0.1% SDS and 1 mM PMSF, following treatment with recombinant rat D-DT protein (Aviva Systems Biology) for 24 h. Alternatively, protein was extracted from 1 cm spinal segments at injured site at 0 day, 1 day, 4 days, and 7 days following contusion (n = 6 in each time point). Samples were vortexed for 30 min and centrifuged at 12,000 rpm for 15 min. The supernatants were collected and stored at -20 ℃ for use. Protein concentration of each specimen was measured by the BCA method to maintain the same loads according to the manufacturer’s instructions. Proteins were heated at 95 ℃ for 5 min, and 20 µg of each sample were electrophoretically separated on 10% SDS-PAGE gel, followed by transferring onto a polyvinylidene difluoride (PVDF) membrane. The membrane was blocked with 5% skim milk in Tris-buffered saline containing 0.1% Tween-20 for 1 h, and then an overnight incubation at 4 ℃ with primary antibodies: D-DT (1:500, Abcam); p65NF-κB (1:1000, CST); ERK (1:1000, CST); p-ERK (1:1000, CST); JNK (1:1000, CST); p-JNK (1:1000, CST); P38 (1:1000, CST); p-P38 (1:1000, CST). After washing 3 times with TBST for 10 min each, the membrane was incubated with secondary antibody goat-anti-mouse HRP or goat-anti-rabbit HRP (1:1000, Beyotime) for 2 h at room temperature. The HRP activity was detected using an ECL kit. The image was scanned with a GS800 Densitometer Scanner (Bio-Rad), and the data were analyzed using PDQuest 7.2.0 software (Bio-Rad). The β-actin (1:5000) was used as an internal control.

### ELISA

Cells or tissue samples were sonicated using the lysis buffer supplemented with a protease inhibitor PMSF as mentioned above. Homogenate was centrifuged at 12,000 rpm for 15 min at 4 ℃, and the supernatant was collected for TNF-α, IL-1β, IL-6 (MULTI SCIENCES), MIF (Elabscience) and D-DT (LMAI) ELISA assay. The concentrations of TNF-α, IL-1β and IL-6 were expressed as pg/ml for the supernatant, while pg/mg for the lysate of the cells or the cord tissues. Plates were read with a multifunctional enzyme marker (Biotek Synergy2) at a 450 nm wavelength.

### Tissue immunofluorescence

The vertebra segments were harvested from six experimental models of each time point, post-fixed, and sectioned. The sections were blocked with 0.01 M PBS containing 3% BSA, 0.1% Triton X-100 and 10% normal goat serum for 1 h at 37 ℃, and incubated overnight at 4 ℃ with primary antibodies: GFAP (1:400, Sigma); OX42 (1:200, Abcam); MBP (1:500, CST); NeuN (1:1000, Abcam); D-DT (1:200, Abcam); S100β (1:400, Abcam); p65NF-κB (1:200, CST). Thereafter, the sections were rinsed with PBS and incubated with the Cy3-labeled goat anti-rabbit IgG (1:400, Abcam) or the Alexa Fluor 488-labeled donkey anti-mouse IgG (1:400, Abcam). Sections were observed under a fluorescence microscope (ZAISS, axio image M2).

### Immunoprecipitation

The primary astrocytes were washed twice with cold phosphate-buffered saline and then extracted with lysis buffer (20 mM Tris-HCl, pH 7.5, 150 mM NaCl, 1 mM EDTA, 1 mM EGTA, 1% Triton X-100, 2.5 mM sodium pyrophosphate, 1 mM β-glycerolphosphate, 1 mM Na_3_VO_4_, 1 mM phenylmethylsulfonyl fluoride and Roche Applied Science’s complete protease inhibitors). Whole cell extracts were centrifuged at 14,000 rpm for 20 min to remove the debris. The proteins in the supernatant were measured using a Protein Assay Kit II (Bio-Rad). For immunoprecipitation analysis, 500 µg of total cell lysates was precleared with protein A plus G-Sepharose before incubation with specific antibodies, followed by addition of protein A plus G-Sepharose. The precipitated proteins were resolved in 2× SDS-PAGE sample buffer and separated by electrophoresis on 12% SDS-PAGE. After being transferred onto a polyvinylidene difluoride membrane (Millipore Corp.), they were incubated with anti-His antibody (1:1000, Proteintech), and further with horseradish peroxidase-conjugated secondary antibody (Santa Cruz).

### Subcellular fractionation

After cell stimulation with 1 µg/ml D-DT recombinant protein for 0 and 24 h, the extraction of nuclear and cytosolic protein from astrocytes was carried out according to the procedure of Nuclear and Cytoplasmic Protein Extraction Kit (Beyotime). Briefly, the cells were washed with ice-cold PBS, followed by lysis in 200 µl cytoplasmic protein extraction agent A, supplemented with 1 mM PMSF on ice for 15 min before a vortex for 5 s. Then the cytoplasmic protein extraction agent B was added, and the mixture was vortexed for 5 s prior to incubation on ice for 1 min. Samples were centrifuged at 16,000*g* at 4 °C for 5 min, and the supernatant (cytosolic fraction) was immediately collected. The pellet (nuclear fraction) was resuspended in nuclear protein extraction agent supplemented with 1 mM PMSF. After 15 to 20 times vortex for 30 min, followed by a centrifugation at 16,000*g* for 10 min, the supernatants containing the nuclear extracts were thus collected. Proteins of nuclear and cytosolic extracts were determined by Western blot. Both β-actin and Histone H3 were used as an internal control of cytoplasmic and nuclear proteins, respectively.

### Sequencing of mRNA

Total RNA of astrocytes following treatment with 1.0 µg/ml recombinant D-DT for 12 h, 24 and 48 h respectively, was extracted using the mirVana miRNA Isolation Kit (Ambion, Austin, TX) according to the manufacturer’s instructions. They were then selected by RNA Purification Beads (Illumina, San Diego, CA), and undergone library construction and RNA-seq analysis. The library was constructed by using the Illumina TruSeq RNA sample Prep Kit v2 and sequenced by the Illumina HiSeq-2000 for 50 cycles. High-quality reads that passed the Illumina quality filters were kept for the sequence analysis.

### Bioinformatics analysis

Differentially expressed mRNA was designated in a criterion of greater or less than twofold changes in comparison with the control. Function of genes was annotated by Blastx against the NCBI database or the AGRIS database with E value threshold of 10^− 5^. Gene ontology (GO) classification was obtained by WEGO via GO id annotated by Perl and R program. Kyoto Encyclopedia of Genes and Genomes (KEGG) pathways were assigned to the sequences using KEGG Automatic Annotation Server (KAAS) online. For all heatmaps, genes were clustered by Jensen-Shannon divergence.

A reconstructed gene network was created using the Ingenuity Pathway Analysis (IPA) Software on the basis of differentially expressed genes to investigate their regulatory pathways and cellular functions.

### Quantitative real-time polymerase chain reaction (Q-PCR)

Total RNA was prepared with Trizol (Sigma) from the cells treated with 50 nM CD74-siRNA (RIBOBIO) using a LipofectamineTM RNAiMAX transfection reagent (Invitrogen) for 24 h. Fluorescence-tagged control Cy3 was used as a marker for evaluation of transfection efficiency. The first-strand cDNA was synthesized using HiScript II Q RT SuperMix for qPCR (+ gDNA wiper) (Vazyme) in a 20 µl reaction system, and diluted at 1:3 before used in assays. The sequence-specific primers were designed and synthesized by Invitrogen (Shanghai, China). Primer pairs for CD74: forward primer 5’-GAC CCG TGA ACT ACC CAC AG-3’, reverse primer 5’-CCA GTG GCT CTT TAG GTG GA-3’; for GAPDH, forward primer 5’-ACA GCA ACA GGG TGG TGG AC-3’, reverse primer 5’-TTT GAG GGT GCA GCG AAC TT-3’. Reactions were performed in a final volume of 10 µl (1 µl cDNA template and 9 µl reaction buffer containing 5 µl of 2 × ChamQ Universal SYBR qPCR Master Mix, 3 µl of RNase free ddH_2_O, and 0.5 µl of anti-sense and sense primers each). Reactions were processed using one initial denaturation cycle at 94 °C for 5 min, followed by 40 cycles of 94 °C for 30 s, 60 °C for 30 s and 72 °C for 30 s. Fluorescence was recorded during each annealing step. At the end of each PCR run, data were automatically analyzed by the system and amplification plots obtained. The expression levels were normalized to an endogenous GAPDH. In addition, a negative control without the first-strand cDNA was also performed.

### Hematoxylin-eosin (HE) staining

The vertebra segments were harvested from six experimental models of each time point, post-fixed, and sectioned. Then the sections were incubated with hematoxylin and eosin, followed by a process according to the standard procedure. Thereafter, the sections were observed under a fluorescence microscope (ZAISS, axio image M2). For quantification of lesion area before or after 4-CPPC treatment, a lesion border with 2000 μm rostral and caudal to the epicenter of the HE-colored sections was selected, and analyzed by NIH ImageJ software. HE area statistics (the area, area fraction expressed as %) were set up (Analyze > Set Measurements) prior to analysis. The lesion area was denoted by 1- HE%.

### Behavioral tests

The hindlimb locomotor function recovery was evaluated using the Basso, Beattie, and Bresnahan (BBB) locomotor scale as previously described [17]. Briefly, after intrathecal injection of 8 µl of 100 mM vehicle or 4-CPPC at 0, 7, 14, and 21 days, three well-trained investigators blind to the study were invited to observe the behavior of rats for 5 min. The BBB score ranged from 0 to 21 according to the rating scale. Every rat had a BBB score of 21 before surgery, and 0 to 1 after a successful SCI.

### Statistical analysis

Statistical analysis used GraphPad Prism 8 software (San Diego, CA, USA). All data were presented as mean ± SD. Comparisons between two groups following a normal distribution were analyzed by two-tailed unpaired Student’s *t* test or the Mann-Whitney test when the distribution was not parametric. Differences between multiple groups were analyzed one­way analysis of variance (ANOVA) or two-way analysis of variance (ANOVA), followed by Dunnett’s or Tukey’s post hoc test. P value < 0.05 was considered statistically significant and are denoted in the figures as *P < 0.05.

## Supplementary Information

**Additional file 1: Fig. S1.** Functional annotations of DEGs in the astrocytes following stimulation with rat recombinant D-DT protein. (a) Primary cultured rat astrocytes of spinal cord stained with GFAP and Hoechst 33342 with purity over 95%. (b) Bar graphs of DEGs following astrocytes stimulation with 1 μg/ml recombinant D-DT protein for 12 h, 24 h, and 48 h, respectively. (c) Integration of DEGs at 12 h, 24 h and 48 h. (d) KEGG enrichment for the DEGs relating to pathways. (e) GO analysis of the DEGs relating to biological processes. Scale bar, 50 μm in (a).

## Data Availability

The datasets used and/or analyzed during the current study are available from the corresponding author on reasonable request.

## Abbreviations

ANOVA: Analysis of variance
CNS: Central nervous system
D-DT: D-dopachrome tautomerase
MIF: Macrophage migration inhibitory factor
DAMP: Damage-associated molecular pattern
PRRs: Pattern recognition receptors
EAE: Experimental autoimmune encephalomyelitis
NF-κB: Nuclear factor kappa-B
PBS: Phosphate buffered saline
MAPK: Mitogen-activated protein kinases
TLRs: Toll-like receptors
SCI: Spinal cord injury
GFAP: Glial fibrillary acid protein
MBP: Myelin basic protein
ELISA: Enzyme-linked immunosorbent assay
