# Supplementary material for: D-dopachrome tautomerase drives astroglial inflammation via NF-κB signaling following spinal cord injury
Source: Cell Biosci. 2022 Aug 14;12:128. doi: 10.1186/s13578-022-00867-7 (PMC9375920; doi:10.1186/s13578-022-00867-7)
Supplement: Supplementary file 1 — Additional file 1: Fig. S1. Functional annotations of DEGs in the astrocytes following stimulation with rat recombinant D-DT protein. (a) Primary cultured rat astrocytes of spinal cord stained with GFAP and Hoechst 33342 with purity over 95%. (b) Bar graphs of DEGs following astrocytes stimulation with 1 μg/ml recombinant D-DT protein for 12 h, 24 h, and 48 h, respectively. (c) Integration of DEGs at 12 h, 24 h and 48 h. (d) KEGG enrichment for the DEGs relating to pathways. (e) GO analysis of the DEGs relating to biological processes. Scale bar, 50 μm in (a). [file 13578_2022_867_MOESM1_ESM.docx]

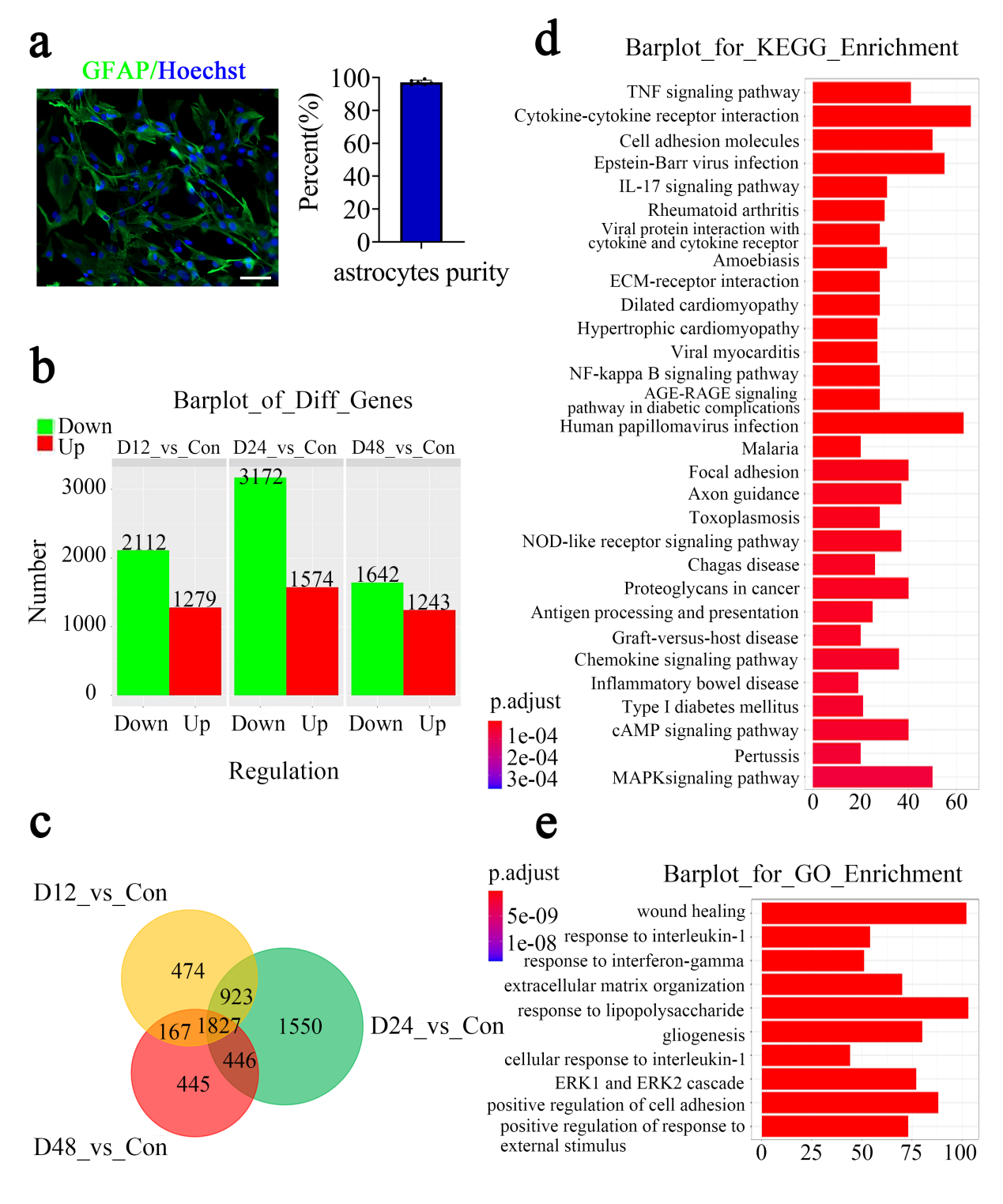


Fig.S1 Functional annotations of DEGs in the astrocytes following stimulation with rat recombinant D-DT protein. (**a**) Primary cultured rat astrocytes of spinal cord stained with GFAP and Hoechst 33342 with purity over 95%. (**b)** Bar graphs of DEGs following astrocytes stimulation with 1 μg/ml recombinant D-DT protein for 12 h, 24 h, and 48 h, respectively. **(c)** Integration of DEGs at 12 h, 24 h and 48 h. **(d)** KEGG enrichment for the DEGs relating to pathways. **(e)** GO analysis of the DEGs relating to biological processes. Scale bar, 50 μm in (**a**).
